# Single passage in mouse organs enhances the survival and spread of *Salmonella enterica*

**DOI:** 10.1098/rsif.2015.0702

**Published:** 2015-12-06

**Authors:** Richard Dybowski, Olivier Restif, Alexandre Goupy, Duncan J. Maskell, Piero Mastroeni, Andrew J. Grant

**Affiliations:** 1 Department of Veterinary Medicine, University of Cambridge, Madingley Road, Cambridge CB3 0ES, UK; 2 ENSTA-ParisTech, 828 Boulevard des Maréchaux, Palaiseau 91120, France

**Keywords:** bacteria, infection, population dynamics, Bayesian

## Abstract

Intravenous inoculation of *Salmonella enterica* serovar Typhimurium into mice is a prime experimental model of invasive salmonellosis. The use of wild-type isogenic tagged strains (WITS) in this system has revealed that bacteria undergo independent bottlenecks in the liver and spleen before establishing a systemic infection. We recently showed that those bacteria that survived the bottleneck exhibited enhanced growth when transferred to naive mice. In this study, we set out to disentangle the components of this *in vivo* adaptation by inoculating mice with WITS grown either *in vitro* or *in vivo*. We developed an original method to estimate the replication and killing rates of bacteria from experimental data, which involved solving the probability-generating function of a non-homogeneous birth–death–immigration process. This revealed a low initial mortality in bacteria obtained from a donor animal. Next, an analysis of WITS distributions in the livers and spleens of recipient animals indicated that *in vivo*-passaged bacteria started spreading between organs earlier than *in vitro*-grown bacteria. These results further our understanding of the influence of passage in a host on the fitness and virulence of *Salmonella enterica* and represent an advance in the power of investigation on the patterns and mechanisms of host–pathogen interactions.

## 1. Introduction

*Salmonella enterica* is a facultative intracellular pathogen capable of causing a spectrum of diseases in humans and other animals. The cumulative global death toll from non-typhoidal *Salmonella* (NTS) gastroenteritis, NTS bacteraemia and typhoid fever is substantial [1]. Current measures to control *S. enterica* infections are suboptimal, and the increasing prevalence of multidrug-resistant strains threatens to limit treatment options [2]. Consequently, there is a need to develop new therapeutic interventions. Experimental infection of mice with *S. enterica* serovar Typhimurium remains an important source of information about the *in vivo* dynamics of infection for both enteric and systemic salmonelloses. Variations in microbial loads in the organs of animals can be quantified post-mortem by plating homogenized tissues on solid culture medium, and counting the numbers of colony-forming units (CFUs) after incubation. While this method provides accurate estimates of the net growth rates of bacterial populations, it bears no information about the respective rates of the underlying processes of bacterial replication, death and migration. For this purpose, various experimental methods for tracking subpopulations of bacteria have been developed [3]. In particular, the use of wild-type isogenic tagged strains (WITS) has enabled a detailed analysis of the bottlenecks undergone by bacterial populations during the course of infection [4,5]. Libraries of WITS are constructed by inserting specific 40 base pair-long oligonucleotides into a non-coding region of the bacterial chromosome. As a result, within a library, all WITS are phenotypically identical, but they can be identified by quantitative PCR. As this allows the quantification of multiple WITS in a mixed culture, it is possible to compare the neutral genetic diversity in mice inoculated with the same mixture of WITS. In particular, we recently demonstrated key differences in the killing and spread of *S.* Typhimurium following immunization of mice with either live or killed vaccines [6].

All WITS experiments consist of infecting mice with a known mixture of tagged wild-type strains and, after a suitable time, recovering the live bacteria from the tissues of interest. The bacteria are then plated for enumeration of CFUs and processed by quantitative PCR (qPCR) in order to assess the relative abundance of the WITS. A critical step in the analysis of these data is the use of mechanistic mathematical models that relate the bacterial numbers and WITS composition to demographic parameters: replication rates, death rates and migration rates. Although the population dynamics of bacteria in single organs can be described with simple stochastic models [4,5], statistical inference on model parameters can rapidly become intractable when movements between multiple compartments are accounted for [6].

Another common point to most published studies of *S. enterica* in mice—and more generally of any bacterial pathogen in animal models—is that the bacteria in the inoculum have been grown *in vitro*. This may result in genetic or epigenetic differences with bacteria that would enter the host via natural routes. Our seminal WITS study [4] showed that *in vitro*-grown *S.* Typhimurium undergoes high mortality upon entering the liver and spleen; but after a few hours, a drop in bactericidal activity allows bacteria to grow exponentially. Although we showed that the initial control is mediated by the host's production of reactive oxygen intermediates [4], it is not clear whether the subsequent shift in dynamics is due to bacterial adaptation. In order to better understand the infection dynamics of *in vivo*-passaged bacteria, we recently compared the dynamics of *S.* Typhimurium colonization in the organs of mice following inoculation with either standard *in vitro*-grown bacteria or bacteria freshly extracted from the organs of infected mice [7]. We found that bacteria transferred after spending between 0.5 and 24 h in the donor host grew faster in the recipient host than *in vitro*-grown bacteria. There was however no apparent change in the initial drop in total bacterial numbers (first 6 h), leading to the hypothesis that *in vivo* adaptation did not make *S.* Typhimurium resistant to the early bactericidal activity.

**Figure 1.**
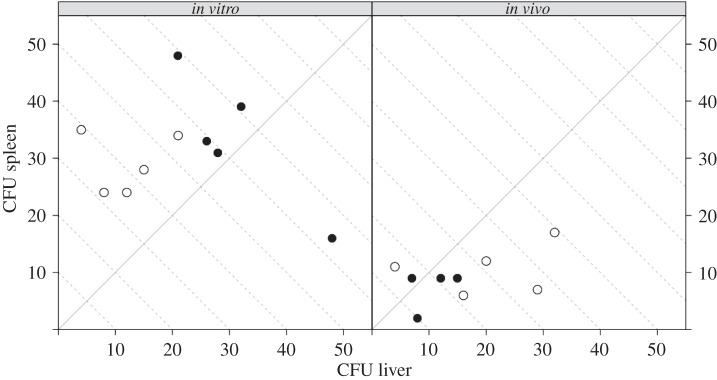
Paired numbers of bacteria (CFU) recovered from the livers and spleens of mice at 0.5 h (filled circles) and 6 h (open circles) after inoculation with *S.* Typhimurium WITS grown *in vitro* (left panel) or *in vivo* (right panel); each dot represents one animal. The dashed lines are isoclines for the total number of CFU per animal.

In order to unravel the differences between the kinetics of *in vitro*-grown and *in vivo*-adapted *S.* Typhimurium, we repeated the transfer experiments from [7] using WITS. More specifically, our objective was to answer two questions: does *in vivo* adaptation affect the initial rates of bacterial replication and death in the liver and spleen? Do *in vivo*-adapted bacteria start moving between the liver and spleen earlier than *in vitro*-grown bacteria? We inoculated groups of mice intravenously with inocula comprising of either an even mixture of eight *S.* Typhimurium WITS grown *in vitro*, or an even mixture of eight WITS, each of them recovered from the spleen of a donor mouse infected with that single WITS. Organs (liver and spleen) of recipient mice were harvested at 0.5, 6, 24, 48 and 72 h post-inoculation (p.i.), live bacteria from each organ were enumerated on agar plates (figure 1), and the WITS composition determined by qPCR. The early dynamics of infection in each organ were modelled as a continuous-time Markovian process, with transition probabilities governed by three rates: immigration, replication and death. We then estimated the parameters of this model with respect to the experimental observations at 0.5 and 6 h p.i. using Bayesian statistics. However, instead of resorting to numerical simulation of the dynamic process, as in reference [6], we derived an analytical expression of the probability-generating function (PGF) that led to a faster and more accurate estimation of the likelihood function. A detailed description of the mathematical and computational methods, which contain substantial improvements from [6], is provided in appendix A.

## 2. Results

### 2.1. Early dynamics (0–6 h p.i.)

**Figure 2.**
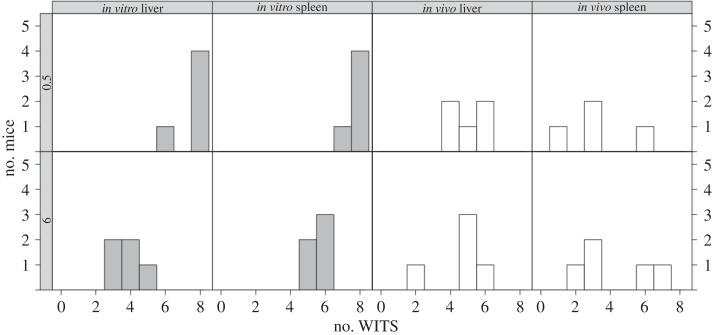
Number of WITS recovered from the livers and spleens of mice in each experimental group at 0.5 h p.i. (top row) and 6 h p.i. (bottom row). Each panel is a histogram representing five mice.

Mice inoculated with *in vitro*-grown *S.* Typhimurium received on average 135 bacteria (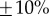). After 30 min, we recovered on average 64 CFU from the organs, equally split between the liver and spleen (resp. 31 and 33 CFU on average, *n* = 5 mice). Within 6 h, the average bacterial loads had dropped to 12 in the liver and 29 in the spleen. All eight WITS were recovered from most organs after 30 min (out of five mice, one animal had one WITS missing from its spleen and another animal had two missing from its liver), whereas all organs harvested after 6 h contained three to six WITS (figure 2). In contrast, the average inoculum size of *in vivo*-grown bacteria was around 31 CFU (range 23–40), and we recovered on average 18 CFU after 30 min (60% of which in livers). By 6 h p.i., however, bacterial loads had increased to 20 CFU in livers and 11 CFU in spleens. On average, around five out of eight WITS were recovered from the livers of mice inoculated with *in vivo*-grown bacteria, and under four WITS from the spleens, with no substantial change between 0.5 and 6 h p.i. (figure 2).

We then estimated the parameters of stochastic models of bacterial dynamics relative to the data on WITS frequencies in mouse organs at 0.5 and 6 h p.i. Because individual *S.* Typhimurium bacteria have been shown to form independent foci of infection in mouse organs [8], we modelled the dynamics of a single WITS in a single organ (liver or spleen) governed by immigration from the bloodstream (from a finite inoculum), replication and death. We assumed that replication and death rates remained constant over the period of time considered (6 h).

**Figure 3.**
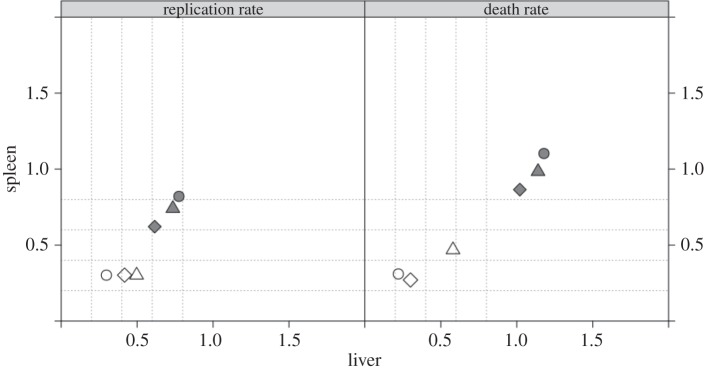
Bayesian estimates for the median replication rate *α* (left panel) and death rate *μ* (right panel) for the *in vitro* (filled symbols) and *in vivo* (open symbols) in the liver (*x*-axis) and spleen (*y*-axis). Three estimates for each parameter in each group and each organ were obtained from three different inoculum sizes.

The results shown in figures 3, 6 and 7 suggest that, within the liver and the spleen, the *per capita* net growth rate during the early period is greater for *in vivo*-grown bacteria than for those grown *in vitro*, with the death rates for the *in vivo* group being less than those for the *in vitro* group.

### 2.2. Expansion phase (6–72 h p.i.)

**Figure 4.**
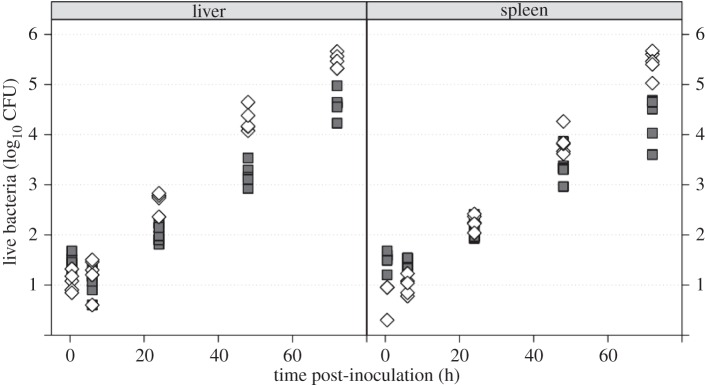
Bacterial load per organ (shown as 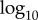 CFU) in mice infected with *in vitro*- (filled symbols) or *in vivo*-grown bacteria (open symbols).

In line with our previous study [7], we found that bacterial loads in livers and spleens increased steadily in both experimental groups from 6 to 72 h p.i. (figure 4). The net growth rate during that period was greater for *in vivo*-grown bacteria (average doubling time 4.6 h) than for *in vitro*-grown bacteria (average doubling time 6.3 h). A linear regression of log(CFU) against time confirmed that the difference in growth rates was statistically significant (
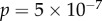
).

**Figure 5.**
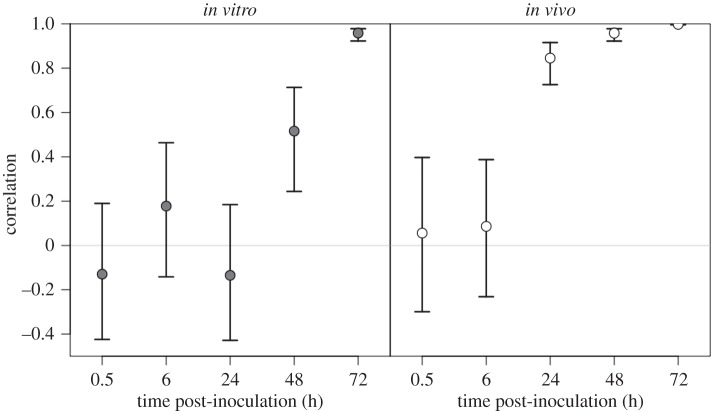
Correlation coefficients (with 95% CIs) of the abundance of the WITS between the liver and spleen within mice, calculated at each time point.

In order to detect spillover of bacteria from the organs back into the bloodstream, we compared the distribution of WITS between the liver and spleen within each mouse. In both experimental groups, the correlation of WITS abundances between the liver and spleen was initially low (and non-significant) for the first 6 h but, by 72 h p.i., the correlation had increased to the point that the bacterial populations in the liver and spleen were virtually indistinguishable (figure 5). However, this increase occurred much more rapidly in recipient mice infected with *in vivo*-grown bacteria than in mice infected with *in vitro*-grown bacteria. This indicates that spillover started between 6 and 24 h p.i. in the former group and between 24 and 48 h p.i. in the latter group. It is worth noting that, by 24 h p.i., the total bacterial loads in four out of five mice infected with *in vivo*-grown bacteria had exceeded the bacterial loads in their counterparts (figure 4).

## 3. Discussion

These results cast a new light on the dynamics of bacterial infection inside hosts. By combining experiments with tagged strains, mathematical models and statistical analysis, we have unravelled two effects of the adaptation of *S.* Typhimurium to *in vivo* growth. Following their transfer from infected animals to naive animals, bacteria were not only able to survive the initial bottleneck better than *in vitro*-grown bacteria, but they also started their systemic spread much earlier (probably 24 h earlier). In particular, we have produced strong evidence against our previous hypothesis that *in vivo* adaptation had no effect on the initial killing of bacteria upon entering the organs [7]. Instead, we suggest that combined reductions in the replication and death of bacteria in the first 6 h of infection underlie variations in total bacterial numbers similar to those observed in mice infected with *in vitro*-grown bacteria.

Although the artificial transfer of bacteria from the organs of a donor mouse to the bloodstream of a recipient animal bypasses key steps in the natural route of transmission of a food-borne pathogen, our findings highlight potential pitfalls in experimental models of infection that use *in vitro*-grown bacteria. Whether *S. enterica* going through oral–faecal transmission would exhibit the same adaptations as our *in vivo*-grown strains is not known at this point, but it would be legitimate to expect discrepancies with *in vitro*-grown bacteria. However, the passage protocol that we followed could bear some resemblance with other routes of infection with *S. enterica* occurring naturally. Contamination of open wounds with *S. enterica* is a public health concern in developing countries, and bacterial contamination of blood products, albeit rare, remains a source of deadly *S. enterica* infection [9].

This study illustrated the benefit of adopting the Bayesian approach to data analysis. In particular, estimation of the posterior probability distributions for the parameters of the birth–death–immigration model has allowed the uncertainty in the parameter values to be estimated. This is in contrast to the maximum-likelihood approach to parameter estimation, which focuses on the estimation of a single value for a parameter.

## 4. Material and methods

### 4.1. Experimental procedures

#### 4.1.1. Bacterial strains and growth conditions

We used *S. enterica* serovar Typhimurium WITS strains 1, 2, 11, 13, 17, 19, 20 and 21 which have been described previously [4]. Briefly, strains were constructed by inserting 40 bp signature tags and a kanamycin resistance cassette between the *malXY* pseudogenes of *S.* Typhimurium JH3016 [10], a gfp^+^ derivative of wild-type virulent SL1344, which has an LD_50_ by the intravenous (i.v.) route of under 20 CFU for innately susceptible mice [11]. Bacterial cultures for infection were grown from single colonies in 10 ml Luria–Bertani (LB) broth incubated overnight without shaking at 37°C, then diluted in phosphate-buffered saline (PBS) to the appropriate concentration for inoculation.

#### 4.1.2. Animals and ethics

We used female eight to nine week old C57BL/6 wild-type mice (Harlan Olac Ltd), which were infected by i.v. injection of bacterial suspensions in a volume of 0.2 ml, and killed up to 72 h p.i. by cervical dislocation. All animals were handled in strict accordance with good animal practice as defined by the relevant international (Directive of the European Parliament and of the Council on the protection of animals used for scientific purposes, Brussels 543/5) and local (Department of Veterinary Medicine, University of Cambridge) animal welfare guidelines.

#### 4.1.3. Generation and transfer of *in vivo*-grown wild-type isogenic tagged strains

To generate the *in vivo*-grown WITS, eight C57BL/6 mice were inoculated i.v. with around 10^4^ CFU of *S.* Typhimurium each mouse receiving a different WITS strain. The mice were killed 72 h p.i. by cervical dislocation, and their spleens were removed aseptically. Each spleen was homogenized using an Ultra-Turrax T25 blender in 5 ml of distilled water. About 1.163 ml of each organ homogenate (9.3 ml total) was added to 30.7 ml of PBS which was further diluted by 10-fold serial dilutions in PBS prior to i.v. inoculation. The bacterial loads in the spleens ranged from 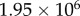 to 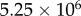 CFU. The transfer of bacteria to the first recipient animal was completed in less than 5 min from the death of the donors.

#### 4.1.4. Enumeration and recovery of viable *Salmonella* in the tissues

Twenty-five recipient mice were inoculated with an even mixture of the eight *in vitro*-grown WITS; the average inoculum size was 135 CFU. Another 25 mice were inoculated with an even mixture of the eight *in vivo*-grown WITS; the average inoculum dose was 31 CFU. At each time point (0.5, 6, 24, 48 and 72 h p.i.), five mice from each experimental group were taken at random and were killed by cervical dislocation. Their livers and spleens were aseptically removed and homogenized separately in 5 ml sterile water using a Colworth Stomacher 80. If required, the resulting homogenate was diluted in a 10-fold series in PBS, and LB agar plates were used to enumerate viable bacteria. Entire organ homogenates in 1 ml aliquots were inoculated onto the surface of 90 mm agar plates. After an overnight incubation at 37°C, colonies were enumerated and total bacteria harvested from the plates by washing with 2 ml PBS. Bacteria were thoroughly mixed by vortexting, harvested by centrifugation and stored at 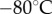 prior to DNA extraction.

#### 4.1.5. Determination of wild-type isogenic tagged strains proportions in bacterial samples by qPCR

DNA was prepared from aliquots of bacterial samples using a DNeasy blood and tissue kit (Qiagen). DNA concentration was determined using a NanoDrop 1000 spectrophotometer (Thermo Scientific). Approximately 10^6^ total genome copies were analysed for the relative proportion of each WITS by qPCR on a Rotor-Gene Q (Qiagen). Duplicate reactions were performed for each sample with primer pairs specific for each WITS in separate 20 µl reactions (primers; table 1). Reactions contained 10 µl of QuantiTect^®^ SYBR^®^ Green PCR kit reagent (Qiagen), 1 µM each primer, 4 µl sample and DNase-free water to 20 µl. Reaction conditions were: 95°C for 15 min, 35 cycles of 94°C for 15 s, 61°C for 30 s and 72°C for 20 s. The copy number of each WITS genome in the sample was determined by reference to standard curves for each primer pair. It was not possible to perform a full standard curve for each primer pair on every rotor; however, individual standards were included on each rotor run to ensure that the values obtained were in the range expected. Standard curves were generated for each batch of PCR reagents by performing qPCRs in duplicate on four separate dilution series of known concentrations of WITS genomic DNA.

**Table 1.** Primers used for qPCR.

primer	tag	sequence 5′ to 3′
ajg497	1	acgacaccactccacaccta
ajg498	2	acccgcaataccaacaactc
ajg503	11	atcccacacactcgatctca
ajg504	13	gctaaagacacccctcactca
ajg507	17	tcaccagcccaccccctca
ajg509	19	gcactatccagccccataac
ajg510	20	acctaactataccgccatcc
ajg511	21	acaaccaccgatcactctcc
ajg520	common	cacggaaaacatcgtgagtc

### 4.2. The early-dynamics model and its parameters

During the early period (0–6 h p.i.), it is assumed that the only events that take place in the liver are the following

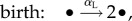


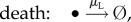


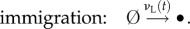


where *α* is the birth rate, *μ* the death rate and 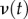 is the rate at which new bacteria feed into the liver from the blood at time *t*. A similar set of parameters exist for the spleen. No emigration of bacteria from the liver and spleen to the blood takes place during the early period. The master equation for this branching process is (with subscript ‘L’ omitted)
4.1
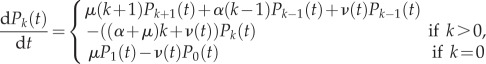
where 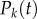 is the probability of having *k* bacteria present at time *t*.

We can derive an expression for 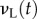 in terms *t* as follows. First, the rate with which the expected value of 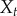 in the blood, 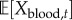, decreases can be expressed as

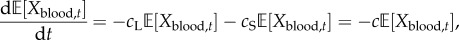
(i.e. 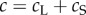) where 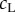 and 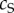 are the rate constants for bacteria moving from the blood to the liver and spleen, respectively; consequently,
4.2
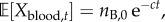
where 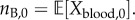 We ignore bacterial replication and death in the blood, on the basis that bacteria are known to reside there for a very short period of time (which we checked *a posteriori* with our parameter estimates). Given also the uncertainty in inoculum sizes and the lack of data on bacterial loads in the blood, it appeared very unlikely we would be able to recover any information on the values of additional parameters from the data. The rate 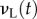 with which bacteria move from the blood to the liver at time *t* is proportional to 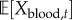 with rate constant 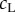,

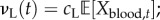
therefore, from (4.2),
4.3
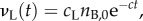
from which we have that 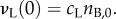 If we let 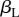 denote 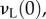 then (4.3) can be rewritten as
4.4
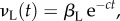
where 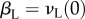 and *c* is an immigration constant. We assume that, for the *w*th WITS, 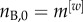.

An analogous case exists for the spleen, and we will use *θ* to represent the vector of parameters for both liver and spleen: 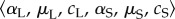.

#### 4.2.1. Data

Data were provided from the mouse experiments using *S. enterica* WITS grown *in vitro* or *in vivo*. The observed data were not the number of WITS *n*, but the corresponding number *u* of CFU; however, for the early-dynamics model, we have used *u* as a proxy for *n*.

For each of the *in vitro* and *in vivo* groups, eight WITS were present in the inocula, and the number *u* of CFU (and thus the number of WITS *n*) present in the liver and spleen 0.5 h and 6 h p.i. were recorded. Five mice were used for each time point.

Let 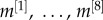 denote the frequencies of the eight WITS injected. If 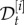 denotes the liver and spleen WITS frequencies from the *i*th mouse for time point *t* following inoculation

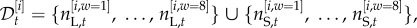
where 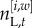 is the frequency of the *w*th WITS present in the liver of the *i*th mouse for time point *t*, then the total data 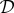 across all mice and time points is

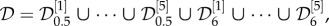
for both the *in vitro* and *in vivo* groups. For each group, there are three estimates of 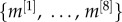.

#### 4.2.2. Parameter estimation

Parameters *θ* for both the *in vitro*- and *in vivo*-grown *S.* Typhimurium can be estimated using Bayesian inference. More precisely, we can estimate the posterior distribution 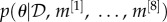 via the relationship
4.5
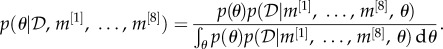


As the mice and WITS are independent of each other, the likelihood 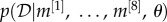 can be factorized as follows
4.6
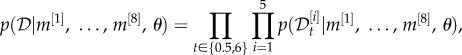
where
4.7
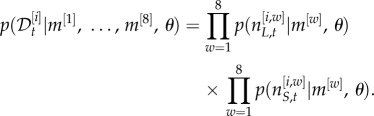
Consequently, determining the posterior probability distribution requires the estimation of 
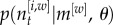
 for each 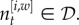 This is described in appendix A.

A robust method for the estimation of the denominator of (4.5) is Markov chain Monte Carlo (MCMC)-based *nested sampling* [12]. Here, the multivariate integral in the denominator of (4.5) is equated to the univariate integral 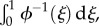 where 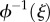 is that likelihood *λ* such that 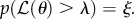 In contrast to the multivariate integral, the univariate integral can be readily estimated by standard numerical methods.

Nested sampling is a sequential process. Starting with a population of particles 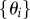 drawn from the prior distribution 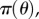 the point 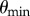 with the smallest likelihood 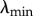 is recorded along with the associated probability 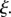 Point 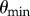 is then replaced by a new point drawn randomly (via MCMC) from the restricted prior 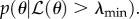 As this process is repeated, the population of points moves progressively higher in likelihood, and the associated restricted priors are nested within each other. The resulting sequence of points 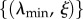 produces the plot required for 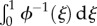.

A drawback of the original version of nested sampling is that it will underestimate the integral if a likelihood function is multimodal. Feroz *et al.* [13] developed a version of nested sampling that can cope with multimodal likelihood functions, but Brewer *et al.* [14] designed a computationally more eloquent approach to this problem called diffusive nested sampling.

**Figure 6.**
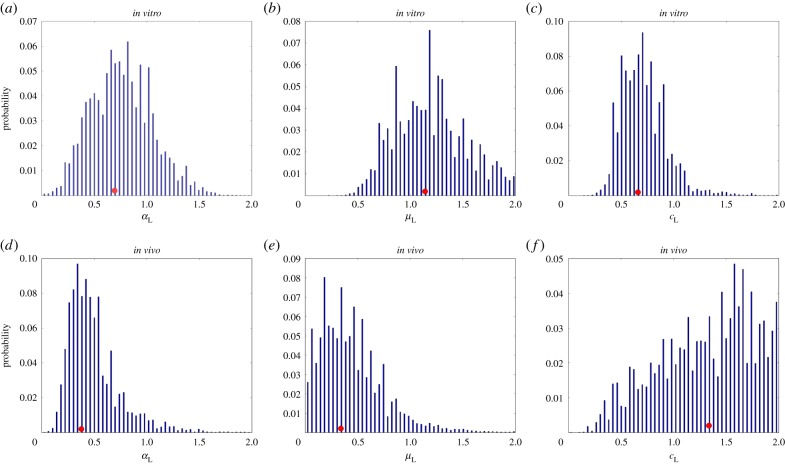
Estimated posterior distributions from the *in vitro* and *in vivo* groups with respect to the liver: (*a,d*) for 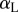 (AUC 0.763); (*b,e*) for 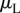 (AUC 0.947); (*c,f*) for 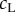 (AUC 0.130). Red dots indicate the positions of the medians.

Rather than confining sampling to a succession of nested restricted priors, *diffusive nested sampling* uses one or more particles to explore a mixture of nested priors, with each successive distribution occupying about 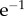 times the enclosed prior mass of the previous distribution. This not only allows lower (earlier) levels to be resampled to improve accuracy, but also allows sampling across multimodal likelihood functions. We performed diffusive nested sampling with 10 000 iterations of a single particle and a maximum of 30 nested levels. For the sake of computational expediency, parameter space was restricted to [0, 2] for each parameter. The uniform prior was used. This parameter space was sufficiently large to illustrate the differences of interest between the posterior distributions in spite of the truncation of 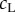 in figure 6*f*.

In order to monitor the progress of the estimation of 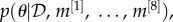 posterior distributions based on subsets of 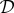 were used: 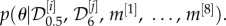 These distributions, computed from likelihood 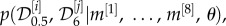 required less time to compute but could be estimated in parallel to each other and then combined as described in appendix A.

**Figure 7.**
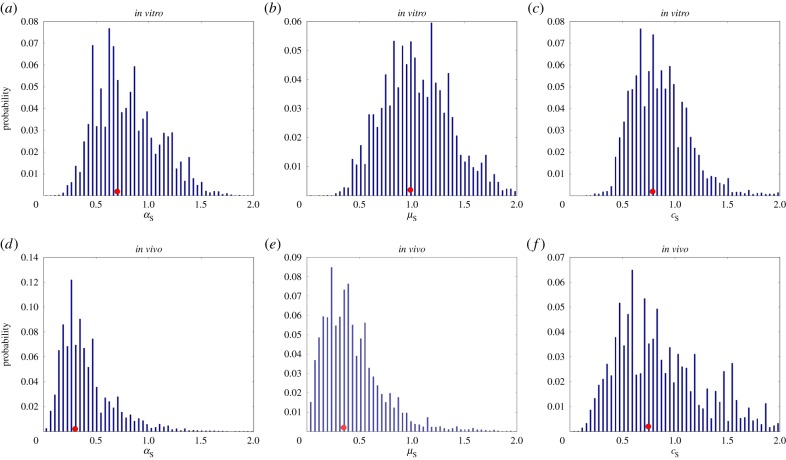
Estimated posterior distributions from the *in vitro* and *in vivo* groups with respect to the spleen: (*a,d*) for 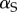 (AUC=0.846); (*b,e*) for 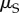 (AUC=0.919); (*c,f*) for 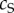 (AUC=0.516). Red dots indicate the positions of the medians.

**Figure 8.**
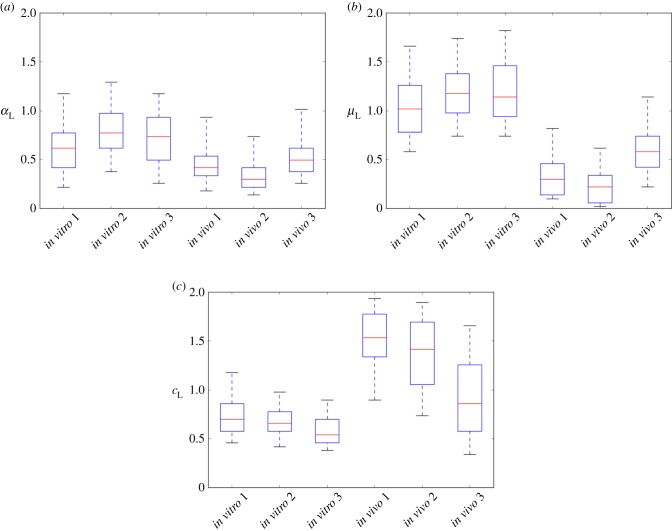
Box plots of the component distributions used for the posterior distributions for (*a*) 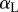, (*b*) 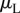 and (*c*) 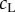 shown in figure 6. The associated number of CFUs in the inocula (all WITS combined) are 124 (*in vitro* 1), 130 (*in vitro* 2), 149 (*in vitro* 3), 23 (*in vivo* 1), 26 (*In vivo* 2) and 40 (*in vivo* 3). The whiskers correspond to the 5% and 95% for the component distributions.

**Figure 9.**
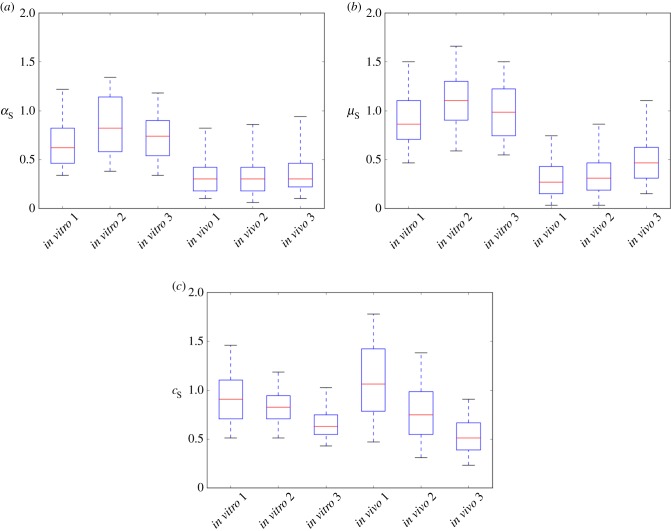
Box plots of the component distributions used for the posterior distributions for (*a*) 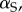 (*b*) 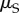 and (*c*) 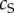 shown in figure 7.

The resulting posterior probability distributions for parameters 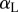, 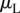, 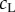, 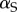, 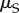 and 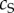 associated with the *in vitro* and *in vivo* groups are shown in figures 6 and 7. Posterior 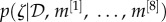 for parameter 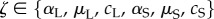 was produced by averaging the posteriors obtained with respect to the three inoculum sizes used for each group (figures 8 and 9). Separation between the *in vitro* and *in vivo* distributions for parameter *ζ* is measured by AUC, which is equal to the probability that *ζ* randomly chosen from the *in vivo* distribution will be less than *ζ* randomly chosen from the *in vitro* distribution.

Kaiser *et al.* [15] have also modelled birth–death–immigration in order to estimate parameters but they used a more simplified model regarding immigration. In contrast, we allowed for the fact that immigration is inhomogeneous as there is a finite number of bacteria immigrating from the bloodstream into the organs. Furthermore, their parameters were estimated using maximum-likelihood without taking into account parameter uncertainties.

Table 2 lists the resulting mean values for the parameters contained in *θ* according to 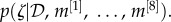

**Table 2.** Mean values and 95% credible intervals (highest probability density intervals) for parameters 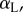 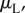 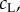 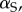 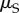 and 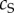 associated with the *in vitro* and *in vivo* groups. Values are restricted to the interval [0, 2] for each parameter. Uniform prior distributions over [0, 2] were used for every parameter.

		mean and 95% HPD interval	
parameter	meaning	*in vitro*	*in vivo*
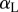	birth rate in liver	0.758 (0.10–1.25)	0.486 (0.10–0.97)
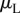	death rate in liver	1.187 (0.58–1.86)	0.433 (0.06–1.06)
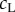	blood-to-liver rate	0.708 (0.34–1.10)	1.302 (0.42–1.97)
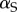	birth rate in spleen	0.793 (0.26–1.38)	0.404 (0.06–1.06)
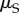	death rate in spleen	1.041 (0.43–1.70)	0.429 (0.06–1.06)
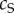	blood-to-spleen rate	0.850 (0.35–1.34)	0.852 (0.15–1.66)

## Supplementary Material

CFU counts and WITS proportions for WITS in vitro in vivo paper.xlsx

## Figures and Tables

**Figure 10.**
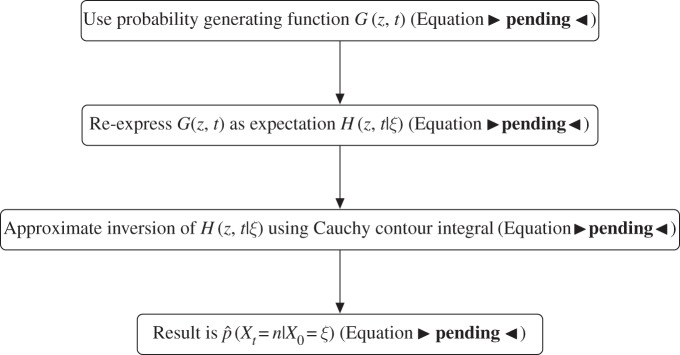
The main steps taken for deriving an expression for the number of bacteria *n* at time *t* starting from a probability generating function. *ξ* is the number of bacteria when *t* = 0, and *z* is a real or complex number.

## Appendix A. The probability of a number of bacteria

The following sections describe the steps taken to deriving an expression for the number of bacteria *n* at time *t* starting from a PGF. Figure 10 highlights the main steps of the derivation.

**A.1. Probability-generating function**

Our approach to the estimation of  has been to use a PGF.

A PGF for the branching process can be defined as

A 1

where *z* is a real or complex number. A virtue of using a PGF is that, in principal, probabilities can be extracted from PGFs by differentiation; for example, in the case of (A 1), we have

A 2

The following partial differential equation can be derived from (A 1) (theorem A.2):

A 3

If there is no immigration (i.e. ) and the branching process begins from a single particle (i.e. ), then (A 3) can be solved [16] to give

A 4

where .

In order to allow for immigration (i.e. ), we consider a single bacterium appearing in the liver from the blood not at time 0 but at some later time *u* > 0. If we denote the PGF for this delayed process by *G*(*z*, *t*, *u*) for , then we can derive an expression for *G*(*z*, *t*, *u*) in a manner analogous to that for (A 4), in which the lower limits for the integrals of (A 4) and definition of function  are replaced with *u*

A 5

where

A 6

According to reference [16], we can write the PGF for when  as follows

A 7

We can solve (A 7) by letting the birth and death rates be constant over time, as follows.

Let  and  then (A 6) becomes

and the integral of (A 5) becomes

This results in (A 5) becoming

A 8

from which we immediately have

A 9

From (A 8) and (4.4), we can write the integral of (A 7) as

A 10

from which we can derive the expression (theorem A.3)

A 11

where  is the Gauss hypergeometric function,

with  denoting the falling factorial:

Finally, substituting (A 9) and (A 11) into (A 7) leads to the relationship

A 12

**A.2. Inversion of the probability-generating function**

Extracting probabilities from PGFs is called *inversion*, and in the case of PGF  we have

A 13

Although inversion of a PGF via differentiation is analytically correct, it can be a formidable task to undertake, depending on the complexity of the PGF. An alternative approach is to use the inversion formula based on the Cauchy contour integral [17],

A 14

where  and *Γ* is a closed contour around 0 in the disc of convergence. If we choose *Γ* to be a circle of radius *r* (0 < *r* < 1) and use the change of variable  then [17]

A 15

A trapezoidal approximation of the integral in (A 15) leads to the following approximation of  [17]

A 16

with error  given by

Here,  is an integer to control the round-off error, and we can set  [18,19]. The error is related to the radius *r* of the disc of convergence for (A 14) by [17,18]

A 17

If *r* is sufficiently small such that (A 17) becomes  then we will have  when  [17].

We can reduce the computation of (A 16) by a factor of 2 by taking the real-valued part of it [17–19]

A 18

In the context of conditional probability  and PGF , (A 18) becomes

A 19

**A.3. Combining posterior probabilities**

Because of the probabilistic independences present within the data, we were able to combine posterior distributions of the form  by application of theorem A.1

where **Φ** is a path-connected subset of parameter space and *k* is the normalization constant. Note that any permutation of {1, 2, 3, 4, 5} could be used for *j*.

**A.4. Accuracy**

The expected number of bacteria at time *t* is given by

but it is also given by

and if  then (theorem A.4)

where

An assessment of the accuracy of using (A 19) can be made by comparing the true expected value based on

with the expectation estimated using those values of  obtained from (A 19)

As an example of such a comparison, the expected values obtained when using , ,  and  were

**Theorem A.1.** *Let *θ* be a point in parameter space and **Φ** a path-connected subset of that space. Let*  *be sets of data that are independent of each other given* *, then*

A 20

*where k is the normalization constant*.

Proof.

A 21

A 22

where  is a normalization constant ensuring that . Now,

A 23

where  is a normalization constant, thus,

A 24

therefore,

▪

Theorem A.2.

A 25

*Proof*. [16, p. 201] Consider the PGF  From the master equation (4.1), we have

and then

Hence, the differential equation

A 26

Note: In the above proof, we can use  in place of *α* and  in place of *μ*.

Lemma A.1.

A 27

*where B*(*a, b*) *is the beta integral and*  *is the hypergeometric function given by*

*Proof*. Because

A 28

we have

A 29

Now

hence

A 30

Theorem A.3.

*Proof*. We have

Consider the variable change  so that  and  then

A 31

where To compute (A 31), we can use the identity (lemma A.1)

as follows

Finally, given that

A 32

we have

▪

Theorem A.4. *The expected number of bacteria at time t is given by*

A 33

*where* *Proof*. From the PGF of the branching process (A 1), we have

A 34

thus

A 35

hence

A 36

Now, if rates *μ* and *α* are assumed to be constant over time, and  is written as  (4.4), then (A 3) can be written as

A 37

in that case

therefore

because *G*(1, *t*) = 1. From (A 35) and (A 36), we can rewrite this as the differential equation

A 38

Solving (A 38) as a first-order differential equation gives

A 39

where *φ* is a constant. This constant can be dealt with as follows. If *ξ* is the initial number of bacteria, then  consequently, setting *t* in (A 39) equal to 0 gives

and the resulting expression for the expected number of bacteria at time *t* is

A 40

where ▪

## References

[1] Mogasale V, Maskery B, Ochiai RL, Lee JS, Mogasale VV, Ramani E, Kim YE, Park JK, Wierzba TF 2014 Burden of typhoid fever in low-income and middle-income countries: a systematic, literature-based update with risk-factor adjustment. The Lancet Glob. Health 2, e570–e580. (10.1016/S2214-109X(14)70301-8) 25304633

[2] Tsolis RM, Xavier MN, Santos RL, Bäumler AJ 2011 How to become a top model: impact of animal experimentation on human *Salmonella* disease research. Infect. Immun. 79, 1806–1814. (10.1128/IAI.01369-10) 21343352 PMC3088149

[3] Crimmins GT, Isberg RR 2012 Analyzing microbial disease at high resolution: following the fate of the bacterium during infection. Curr. Opin. Microbiol. 15, 23–27. Host–microbe interactions: bacteria. See http://www.sciencedirect.com/science/article/pii/S1369527411001883 (10.1016/j.mib.2011.11.005) 22143042 PMC3265638

[4] Grant AJ, Restif O, McKinley TJ, Sheppard M, Maskell DJ, Mastroeni P 2008 Modelling within-host spatiotemporal dynamics of invasive bacterial disease. PLoS Biol. 6, e74 (10.1371/journal.pbio.0060074) 18399718 PMC2288627

[5] Kaiser P, Slack E, Grant AJ, Hardt WD, Regoes RR 2013 Lymph node colonization dynamics after oral *Salmonella* Typhimurium infection in mice. PLoS Pathog. 9, e1003532 (10.1371/journal.ppat.1003532) 24068916 PMC3777876

[6] Coward C, Restif O, Dybowski R, Grant AJ, Maskell DJ, Mastroeni P 2014 The effects of vaccination and immunity on bacterial infection dynamics *in vivo*. PLoS Pathog. 10, e1004359 (10.1371/journal.ppat.1004359) 25233077 PMC4169467

[7] Mastroeni P, Morgan FJE, McKinley TJ, Shawcroft E, Clare S, Maskell DJ, Grant AJ 2011 Enhanced virulence of *Salmonella enterica* serovar Typhimurium after passage through mice. Infect. Immun. 79, 636–643. (10.1128/IAI.00954-10) 21098099 PMC3028859

[8] Sheppard M, Webb C, Heatth F, Mallows V, Emilianus R, Maskell DJ, Mastroeni P 2003 Dynamics of bacterial growth and distribution within the liver during *Salmonella* infection. Cell Microbiol. 5, 593–600. (10.1046/j.1462-5822.2003.00296.x) 12925129

[9] Brecher ME, Hay SN 2005 Bacterial contamination of blood components. Clin. Microbiol. Rev. 18, 195–204. (10.1128/CMR.18.1.195-204.2005) 15653826 PMC544173

[10] Hautefort I, Proença MJ, Hinton JC 2003 Single-copy green fluorescent protein gene fusions allow accurate measurement of *Salmonella* gene expression *in vitro* and during infection of mammalian cells. Appl. Environ. Microbiol. 69, 7480–7491. (10.1128/AEM.69.12.7480-7491.2003) 14660401 PMC310007

[11] Hoiseth SK, Stocker BAD 1981 Aromatic-dependent *Salmonella typhimurium* are non-virulent and effective as live vaccines. Nature 291, 238–239. (10.1038/291238a0) 7015147

[12] Skilling J 2006 Nested sampling for general Bayesian computation. Bayesian Anal. 1, 833–860. (10.1214/06-BA127)

[13] Feroz F, Hobson MP, Bridges M 2009 MultiNest: an efficient and robust Bayesian inference tool for cosmology and particle physics. Mon. Notes Royal Astron. Soc. 398, 1601–1614. (10.1111/j.1365-2966.2009.14548.x)

[14] Brewer BJ, Pártay LB, Csányi G 2011 Diffusive nested sampling. Stat. Comput. 21, 649–656. (10.1007/s11222-010-9198-8)

[15] Kaiser P, Regoes RR, Dolowschiak T, Wotzka SY, Lengefeld J, Slack E, Grant AJ, Ackermann M, Hardt W-D 2014 Cecum lymph node dendritic cells harbor slow-growing bacteria phenotypically tolerant to antibiotic treatment. PLoS Biol. 12, e1001793 (10.1371/journal.pbio.1001793) 24558351 PMC3928039

[16] Lange K 2010 Applied probability. 2nd edn. New York, NY: Springer.

[17] Abate J, Choudhury GL, Whitt W 1999 An introduction to numerical transform inversion and its application to probability models. In Computational probability (ed. Grassman W), pp. 257–323. Boston, MA: Kluwer.

[18] Abate J, Whitt W 1992 Numerical inversion of probability generating functions. Oper. Res. Lett. 12, 245–251. (10.1016/0167-6377(92)90050-D)

[19] Abate J, Whitt W 1992 The Fourier-series method for inverting transforms of probability distributions. Queuing Syst. 10, 5–88. (10.1007/BF01158520)

